# EPA and DHA Inhibit Myogenesis and Downregulate the Expression of Muscle-related Genes in C2C12 Myoblasts

**DOI:** 10.3390/genes10010064

**Published:** 2019-01-18

**Authors:** Jing Zhang, Xin Xu, Yan Liu, Lin Zhang, Jack Odle, Xi Lin, Huiling Zhu, Xiuying Wang, Yulan Liu

**Affiliations:** 1 Hubei Key Laboratory of Animal Nutrition and Feed Science, Hubei Collaborative Innovation Center for Animal Nutrition and Feed Safety, Wuhan Polytechnic University, Wuhan 430023, China; judyzhang1103@126.com (J.Z.); xuxin199400@126.com (X.X.); sharonliuyan@126.com (Y.L.); lynnemumu@126.com (L.Z.); zhuhuiling2004@sina.com (H.Z.); xiuyingdk@foxmail.com (X.W.); 2 Laboratory of Development Nutrition, Department of Animal Science, North Carolina State University, Raleigh, NC 27695, USA; jodle@ncsu.edu (J.O.); xilin@ncsu.edu (X.L.)

**Keywords:** C2C12, proliferation, differentiation, EPA, DHA

## Abstract

This study was conducted to elucidate the biological effects of eicosapentaenoic acid (EPA) and docosahexaenoic acid (DHA) on cell proliferation, differentiation and gene expression in C2C12 myoblasts. C2C12 were treated with various concentrations of EPA or DHA under proliferation and differentiation conditions. Cell viability was analyzed using cell counting kit-8 assays (CCK-8). The Edu assays were performed to analyze cell proliferation. To analyze cell differentiation, the expressions of myogenic marker genes were determined at the transcriptional and translational levels by qRT-PCR, immunoblotting and immunofluorescence. Global gene expression patterns were characterized using RNA-sequencing. Phosphorylation levels of ERK and Akt were examined by immunoblotting. Cell viability and proliferation was significantly inhibited after incubation with EPA (50 and 100 μM) or DHA (100 μM). Both EPA and DHA suppressed C2C12 myoblasts differentiation. RNA-sequencing analysis revealed that some muscle-related genes were significantly downregulated following EPA or DHA (50 μM) treatment, including insulin-like growth factor 2 (IGF-2), troponin T3 (Tnnt3), myoglobin (Mb), myosin light chain phosphorylatable fast skeletal muscle (Mylpf) and myosin heavy polypeptide 3 (Myh3). IGF-2 was crucial for the growth and differentiation of skeletal muscle and could activate the PI3K/Akt and the MAPK/ERK cascade. We found that EPA and DHA (50 μM) decreased the phosphorylation levels of ERK1/2 and Akt in C2C12 myoblasts. Thus, this study suggested that EPA and DHA exerted an inhibitory effect on myoblast proliferation and differentiation and downregulated muscle-related genes expression.

## 1. Introduction

Skeletal muscle, the largest tissue in the body, is important in locomotion and metabolic adaptation. Skeletal muscle formation mainly occurs during fetal development, whereas skeletal muscle hypertrophy occurs after birth [1]. Prenatal myogenesis is a complex process, in which somite-derived myoblasts withdraw from the cell cycle after proliferation and terminally differentiate into myotubes [2]. C2C12 cells derived from the mouse skeletal muscle C2 cell line contain the properties of myoblast progenitor lineage [3]. Thus, they provide a well-established cell model for studying the major steps involved in myogenesis.

Previous in vitro studies have demonstrated that fatty acids can modulate myoblast proliferation and myogenic differentiation. Oleic acid (OA), linoleic acid (LA), γ-linoleic acid (GLA), arachidonic acid (AA) and cis-9, trans-11 conjugated linoleic acid (c9, t11 CLA) exert a proliferative effect on C2C12 myoblasts [4]. Some studies have also shown the stimulatory effects of OA, LA and c9, t11 CLA on skeletal muscle differentiation. Conversely, trans-10, cis-12 conjugated linoleic acid (t10, c12 CLA) inhibits C2C12 myoblast proliferation and differentiation [4,5].

Long chain (n-3) polyunsaturated fatty acids (PUFAs) such as eicosapentaenoic acid (EPA) (20:5n3) and docosahexaenoic acid (DHA) (22:6n3), which are abundant in fish oil, are considered essential fatty acids with an array of health benefits [6,7,8,9]. A large number of studies currently using the C2C12 myotubes model have confirmed that EPA and DHA can prevent muscle atrophy, promote muscle protein synthesis, regulate lipid metabolism and improve insulin resistance in skeletal muscle [10,11,12]. Kamolrat et al. [10] reported that EPA (50 μM) significantly enhanced protein synthesis and reduced protein breakdown in C2C12 myotubes. Capel et al. [11] found that 30 μM DHA prevented insulin resistance in C2C12 myotubes exposed to 500 μM palmitate (PAL). However, the information about the biological effects of EPA and DHA on C2C12 myoblast proliferation and differentiation is limited and inconsistent [12].

The MAPK/ERK and PI3K/Akt pathways are critical signaling cascades in the regulation of skeletal muscle development. The MAPK/ERK pathway plays an important role in the promotion of myoblast proliferation [13,14], while activation of the PI3K/Akt pathway positively regulated myogenic differentiation [15,16]. There is increasing evidence that EPA and DHA modulate ERK and Akt signaling in many different cell types [17,18,19]. EPA and DHA can inhibit lymphocyte proliferation by reducing the phosphorylation of ERK1/2 and Akt [20]. Therefore, we hypothesize that EPA and DHA treatment might affect myogenesis and modulate the phosphorylation of ERK and Akt in C2C12 myoblasts. The objective of the current study was to determine the effects of EPA and DHA supplement on myogenesis process and gene expression in C2C12 myoblasts. Our results will suggest a role for n-3 PUFA-mediated control of skeletal muscle development.

## 2. Materials and Methods

### 2.1. Cell Culture and Fatty Acids Preparation

C2C12 myoblasts were purchased from American Type Culture Collection (ATCC). C2C12 cells were cultured under 5% CO_2_ at 37 °C in growth medium consisting of DMEM (high glucose, glutamine and pyruvate) supplemented with 10% FBS (Gibco) and 1% penicillin/streptomycin (Gibco). To induce differentiation, the medium was changed to differentiation medium containing DMEM (high glucose, glutamine and pyruvate) supplemented with 2% horse serum (Gibco) and 1% penicillin/streptomycin. The fatty acids were dissolved in 100% ethanol and combined with bovine serum albumin (BSA) in a 4:1 molar ration (fatty acid/BSA) [21].

### 2.2. Cell Proliferation Assay

Cells were seeded in a 96-well plate at 2 × 10^3^ cells/well and cultured in a growth medium. The cells, 12 h after plating, were washed twice in PBS and treated with various concentrations (0, 6.25, 12.5, 25, 50 and 100 μM) of EPA or DHA (Sigma-Aldrich, Purity ≥ 98%). Each well contained an equivalent volume of ethanol and the final concentration of ethanol was 0.01%. The CCK-8 assay (Dojindo) was used to quantify proliferating cells at 12, 24, 48 and 72 h after EPA or DHA treatment. Then, 10 μL of CCK-8 reagents was added to the cells for 1h. Absorbance at 450 nm was measured using the SpectraMax M5 microplate spectrophotometer.

### 2.3. Edu Assays

The Edu assay was performed using the BeyoClick™ EdU-488 assay kit (Beyotime, C0071S) according to the manufacturer’s instructions. Briefly, 48 h after the EPA or DHA (50 or 100 μM) treatment, cells were exposed to 10 μM Edu for 2 h. Next, cells were fixed with 4% paraformaldehyde and permeabilized with 0.5% triton X-100. Subsequently, cells were incubated in the Click Additive Solution for 30 min and stained with DAPI. Fluorescence imaging of Edu^+^ cells was obtained with an Olympus Fluoview FV10C-O3 confocal microscope. The cells were further analyzed by calculating the ratio of Edu^+^ cells to the total number of cells.

### 2.4. qRT-PCR Measurement

Total RNA isolation, quantification, cDNA synthesis and RT-qPCR were carried out as previously described [9]. The housekeeping gene β-actin was used as an internal normalization control [22]. The primers used for qRT-PCR were listed in Table 1. All data were analyzed using the 2^−ΔΔ*C*T^ method [23].

### 2.5. Immunoblotting

Immunoblotting was performed as previously described [9], and the antibodies included MHC (MF20; DSHB; 1:1000), myogenin (M-225; Santa Cruz; #sc-576; 1:1000), phosphorylated Akt (serine473; Cell Signaling; #9271; 1:1000), total Akt (Cell Signaling; #9272; 1:1000), phosphorylated ERK1/2 (Thr202/Tyr204; Cell Signaling; #9101; 1:1000); total ERK1/2 (Cell Signaling; #9102; 1:1000) and β-actin (Sigma Aldrich; #A2228; 1:1000).

### 2.6. Immunofluorescence

C2C12 cells treated in 6-well plates were fixed in 4% formaldehyde for 10 min and then washed three times for 10 min each in PBS. The cells were then permeabilized with 0.1% Triton X-100 for 10 min. After blocking with 5% skim milk in PBS, the cells were incubated with the primary antibody MF20 and myogenin (1:40 dilution) for 1 h at 37 °C. The FITC-labeled anti-mouse IgG (Santa Cruz; #sc-2010; 1:5000) was incubated for 1 h at 37 °C. The nuclei of the cells were visualized using DAPI staining. Fluorescence imaging was obtained with Olympus IX73.

### 2.7. RNA-sequencing and Data Analysis

C2C12 cells cultured in the growth medium were treated without (control) or with EPA or DHA (50 μM). There were three biological replicates for each treatment. Total RNA from each sample was isolated 48 h after treatment. The total RNA isolation from each sample was carried out as previously described [9]. cDNA libraries construction and RNA-Sequencing were performed at BGI (Shenzhen, China) according to the manufacturer’s specifications. Briefly, mRNA was enriched from total RNA using oligo (dT) magnetic beads. A fragmentation buffer was used to reduce the mRNA into short fragments. The first strand cDNA was synthesized with random primers using the mRNA fragments as templates. Buffer, dNTPs, RNase H and DNA polymerase I were added to synthesize the second strand cDNA. The double-strand cDNA (dscDNA) was purified with a QiaQuick PCR extraction kit and washed with EB buffer for end repair and 5′ end phosphate. Then two blunt end adaptors were ligated to the dscDNA. The ligation product was nick translated to intact dsDNA followed by PCR amplification. The PCR product was heat denatured, and the single strand DNA was cyclized by splint oligo and DNA ligase to construct cDNA libraries. Finally, RNA-sequencing was performed using the sequencing platform of Complete Genomics. After obtaining the raw reads, the adapter, high content of unknown bases and low-quality reads were removed before downstream analysis to decrease data noise. After filtering, the clean reads were stored as FASTQ format. The fragments per kilobase per million map reads (FPKM) were calculated for each gene to normalize the data. The NOISeq method was used to screen differentially expressed genes (DEGs) between the treatment groups [24]. We screened DEGs according to the following default criteria: Fold change ≥ 2 and diverge probability ≥ 0.8. We applied DAVID 6.8 to perform Gene Ontology (GO) functional analysis and KEGG pathways analysis of DEGs. The enriched GO terms and pathways with *p* value < 0.05. Our data were submitted to the SRA database: https://www.ncbi.nlm.nih.gov/sra/PRJNA491238.

### 2.8. Statistical Analysis

The data are shown as means ± S.D. Differences were tested using ANOVA and the Student’s paired *t*-test. The level of significance was set at *p* < 0.05 for all data analyses.

## 3. Results

### 3.1. Inhibitory Effects of EPA and DHA on C2C12 Myoblast Proliferation

**Figure 1:**
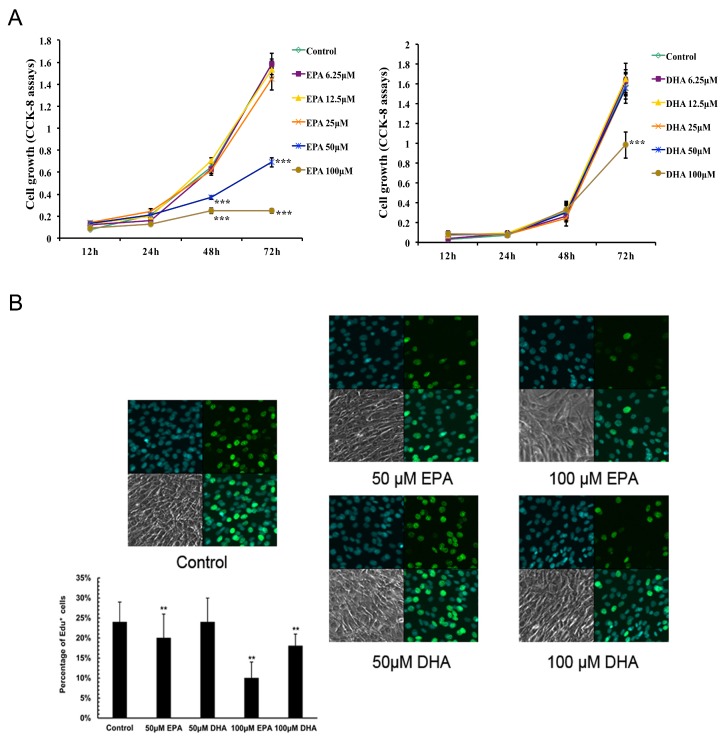
The effects of eicosapentaenoic acid (EPA) and docosahexaenoic acid (DHA) on the viability and proliferation of C2C12 myoblasts. C2C12 myoblasts were treated with EPA or DHA at different concentrations. (**A**) The CCK-8 assay was performed to measure cell viability at 12, 24, 48 and 72 h after EPA or DHA treatment. The *X* axis represents time of treatment. The *Y* axis represents the absorbance determined at 450 nm. The data represent the mean ± S.D. from three independent experiments performed in duplicate. Different from control, *** *p* < 0.001. (**B**) C2C12 myoblasts were treated with EPA or DHA at concentrations of 50 or 100 μM. Cells were stained with Edu. Original magnifications 600×. The percentage of Edu^+^ C2C12 cells was quantified. The data represent the mean ± S.D. (*n* = 6). Different from control, ** *p* < 0.01.

C2C12 myoblasts were treated with varying concentrations of EPA or DHA for 12, 24, 48 and 72 h under standard conditions. We then monitored the treated cells for alterations in viability using the CCK-8 assay. Compared with the control, the inhibitory effect was obvious following treatment with 50 or 100 μM EPA for 48 h and 72 h or with 100 μM DHA for 72 h (Figure 1A) (*p* < 0.001). Furthermore, we performed Edu assays to analyze the effects of EPA or DHA on C2C12 proliferation at the concentration of 50 and 100 μM. Edu staining demonstrated that the Edu^+^ cells were significantly reduced in C2C12 myoblast treated with EPA (50 and 100 μM) and DHA (100 μM) for 48 h compared with that of the control (Figure 1B). These results indicated that EPA inhibited the proliferation of C2C12 myoblast to a greater extent than DHA at the same concentration.

### 3.2. Inhibitory Effects of EPA and DHA on C2C12 Myoblast Differentiation

**Figure 2:**
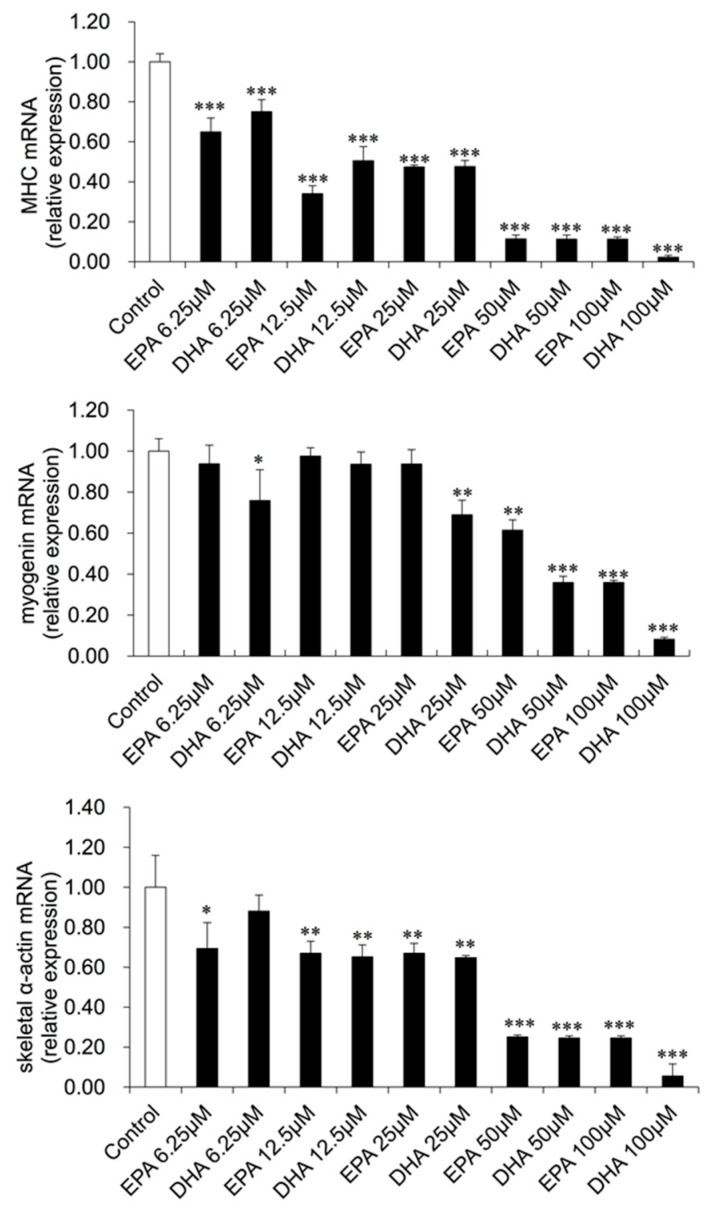
The effects of EPA and DHA on the mRNA expressions of the myogenic marker genes in C2C12 myoblasts. C2C12 myoblasts were cultured in differentiation medium with various concentrations of EPA or DHA for 48 h. qRT-PCR was performed to examine the mRNA expression levels of MHC, myogenin and skeletal α-actin. The data represent the mean ± S.D. from three independent experiments performed in duplicate. Different from control, * *p* < 0.05, ** *p* < 0.01 and *** *p* < 0.001.

Myogenin is a basic helix–loop–helix transcription factor that belongs to the MRF gene family, which can activate myogenic differentiation [2]. During the transition from proliferating myoblasts to terminally differentiated myotubes, muscle-specific contractile protein genes are expressed including MHC, Tnnt and skeletal α-actin [25]. Thus, myogenin, MHC and skeletal α-actin can be used as muscle-specific myogenic markers to determine the extent of myogenesis [22].To further investigate the effects of EPA and DHA on myoblast differentiation, the growth medium was changed to the differentiation medium to induce differentiation, and myoblasts were treated with various concentrations of EPA or DHA for 48 h. qRT-PCR was used to quantify the mRNA abundance of the myogenic marker genes MHC, myogenin and skeletal α-actin at the transcriptional level. As shown in Figure 2, EPA and DHA significantly reduced MHC and skeletal α-actin at the low concentration of 6.25, 12.5 and 25 μM (*p* < 0.01) while the DHA and EPA inhibitory effect on myogenin expressions started at 25 and 50 μM, resepctively (*p* < 0.05), and the inhibitory effect was stronger in cells treated with 50 or 100 μM EPA or DHA (*p* < 0.001).

**Figure 3:**
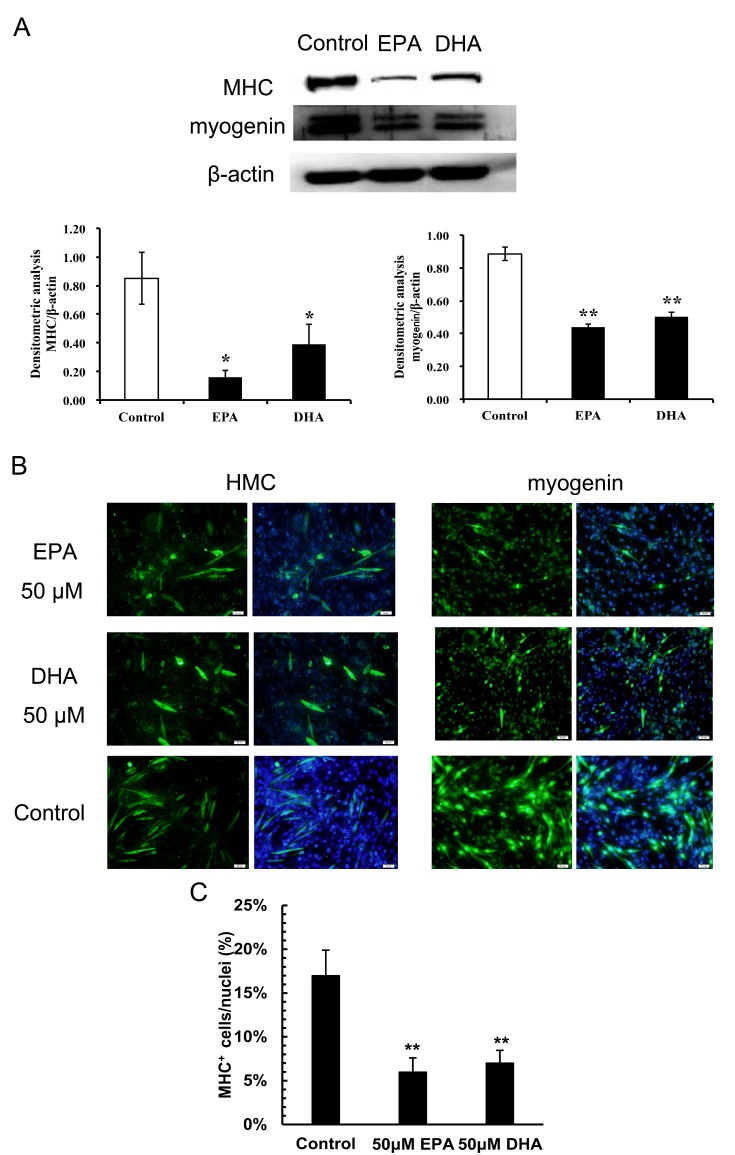
The effects of EPA and DHA on the protein expressions of the myogenic marker genes in C2C12 myoblasts. C2C12 myoblasts were treated without (control) or with EPA or DHA (50 μM) under differentiation conditions for 72 h. (**A**) Immunoblotting experiments and (**B**) immunofluorescence staining were performed to determine the protein levels of MHC and myogenin. For immunoblotting experiments, the data represent the mean ± S.D. from three independent experiments performed in duplicate. Different from control, * *p* < 0.05 and ** *p* < 0.01. (**C**) The percentage of MHC^+^ cells to the total nuclei was quantified. The data represent the mean ± S.D. (*n* = 3). Different from control, ** *p* < 0.01.

In addition, C2C12 myoblasts were then treated with 50 μM EPA or DHA under differentiation conditions for 72 h. Immunoblotting and immunofluorescence staining was used to determine MHC and myogenin at the translational level. Compared with the control, 50 μM EPA and DHA markedly reduced the protein level of myogenin and the number of MHC^+^ cells (Figure 3), suggesting EPA and DHA suppressed the differentiation of C2C12 myoblasts.

### 3.3. Gene Expression Changes in EPA- or DHA-treated C2C12 Cells

**Figure 4:**
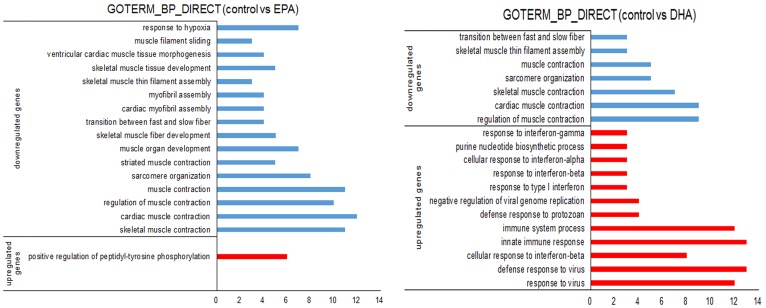
The enriched Gene Ontology (GO) terms of the biological process (BP) form the differentially expressed genes (DEGs) in EPA treatment and DHA treatment. The *X* axis represents the number of DEGs. The *Y* axis represents GO terms.

We found that EPA or DHA (50 μM or 100 μM) significantly inhibited C2C12 myoblast proliferation and reduced the mRNA expressions of the myogenic marker genes at 48 h. Therefore, we further analyzed gene expression changes in C2C12 myoblasts after the EPA or DHA (50 μM) treatment for 48 h by using RNA sequencing (RNA-seq). RNA-seq analysis revealed a large number of mRNA changes in response to EPA or DHA treatment (Figure S1). Compared with the control, we identified 130 (62 upregulated and 68 downregulated genes) and 204 (69 upregulated and 135 downregulated genes) DEGs in DHA and EPA treatment respectively (Table S1). To explore the biological meaning of the DEGs, we employed the DAVID Functional Annotation Tool (6.8) to perform a functional analysis. The upregulated DEGs from EPA treatment were assigned to the positive regulation of peptidyl-tyrosine phosphorylation, while the majority of the upregulated DEGs from the DHA treatment were associated with the immune biological process, such as response to interferon-gamma/alpha/beta, immune system process and response to virus (Figure 4). However, the most enriched GO terms of the downregulated DEGs from the EPA or DHA treatment were related to muscle development, such as skeletal muscle contraction, sarcomere organization, transition between fast and slow fiber, skeletal muscle fiber development and skeletal muscle thin filament assembly (Figure 4). In addition, we performed a pathway analysis of the DEGs, but none of the skeletal muscle development related pathways were enriched (Figure S1).

**Figure 5:**
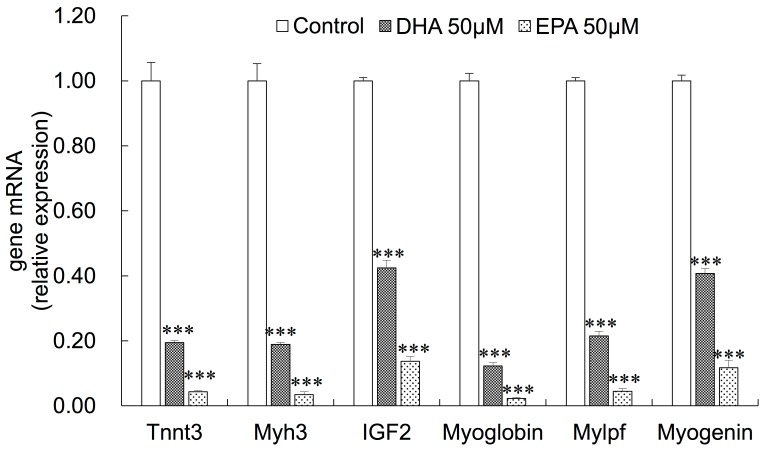
qRT-PCR results of selected genes. The data represent the mean ± S.D. from three independent experiments performed in duplicate. Different from control, *** *p* < 0.001.

In addition, we found that some of the muscle-specific genes were significantly downregulated in EPA- or DHA-treated myoblasts, including troponin T1 (Tnnt1), troponin T3 (Tnnt3), troponin C1 (Tnnc1), troponin C2 (Tnnc2), myosin light chain phosphorylatable fast skeletal muscle (Mylpf), myoglobin (Mb), myosin heavy polypeptide 3 (Myh3), myosin heavy polypeptide 1 (Myh1), myosin light polypeptide 4 (Myl4) and myogenin (Table S1). In addition, EPA and DHA significantly reduced IGF-2 mRNA expression. The downregulation of these muscle-related genes might be related to the inhibitory effects of EPA and DHA on C2C12 myoblasts differentiation. To verify the RNA-seq data, we confirmed six genes (myogenin, Tnnt3, Mylpf, Mb, Myh3 and IGF2) using qRT-PCR (Figure 5).

### 3.4. Inhibitory Effects of EPA and DHA on the Phosphorylation of ERK1/2 and Akt

**Figure 6:**
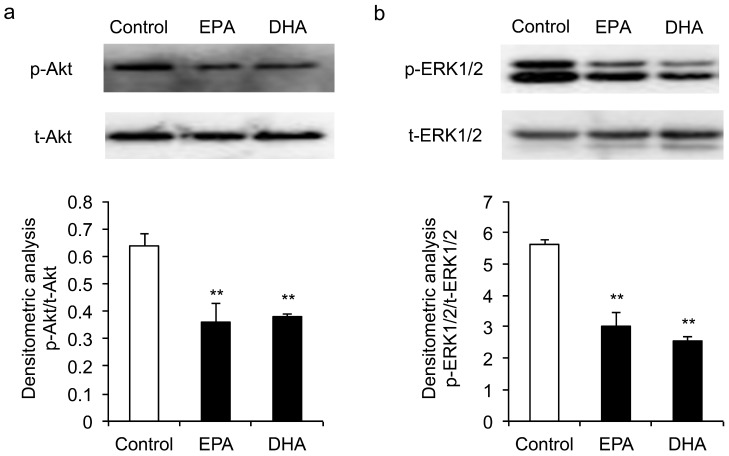
The effect of EPA and DHA on the phosphorylation levels of (**A**) Akt and (**B**) ERK1/2 in C2C12 myoblasts. The data represent the mean ± S.D. from three independent experiments performed in duplicate. Different from control, ** *p* < 0.01.

The MAPK/ERK1/2 and PI3K/Akt pathways have been implicated in regulating the proliferation and differentiation of skeletal muscle myoblasts. We examined the phosphorylation of Akt and ERK1/2 in C2C12 myoblasts after treatment with 50 μM EPA or DHA at 1 h. As shown in Figure 6, EPA and DHA significantly reduced the phosphorylated protein levels of ERK1/2 and Akt, suggesting the ERK1/2 and PI3K/Akt signaling might be involved in the regulation of changes caused by EPA or DHA.

## 4. Discussion

Recently, maternal nutrition guidelines stressed the importance of n-3 PUFAs supplementation during pregnancy. This is partially because EPA and DHA are beneficial for fetal development, including neuronal, retinal and immune functions [26]. However, over-supplementation of n-3 PUFAs resulted in growth deficiencies by causing a form of nutritional toxicity [27]. During fetal development, skeletal muscle formation can also be modulated by maternal nutrition [28]. However, information on the roles of EPA and DHA on skeletal muscle development is limited. C2C12 myoblasts can be utilized as an in vitro model to study the fetal muscle development. Based on this, we investigated the effects of EPA and DHA on C2C12 myoblasts proliferation and differentiation in the present study. In addition, to our knowledge, this study is the first to examine gene expression profiles in C2C12 myoblasts treated with EPA and DHA, which furthers our understanding of n-3 PUFAs function in the regulation of skeletal muscle development.

Lee et al. [4] treated C2C12 myoblasts with various concentrations of fatty acids (0.1, 1 or 10 μM) for 48 h during proliferation. They found that DHA stimulated cell proliferation at 10 μM, but EPA had no obvious effect on the proliferation. In this present study, we chose the concentrations of EPA or DHA ranging from 6.25 to 100 μM and treated C2C12 for 72 h in the proliferation condition. We observed that the viability of C2C12 myoblasts was significantly inhibited from 48 to 72 h by EPA at 50 and 100 μM. By comparison, the growth-inhibiting effect of DHA was observed at 72 h at a concentration of 100 μM while when myoblasts were treated with EPA or DHA at the low concentration (6.25, 12.5 and 25 μM), the viability-inhibiting was not detected within 72 h. The Edu assays also demonstrated that EPA and DHA could inhibit C2C12 myoblasts proliferation at high concentrations. Our results partly corroborate the findings of Peng et al. [29], who have observed that DHA and EPA (50 or 100 μM) significantly inhibited C2C12 myoblasts proliferation within 24 h but that 50 μM linolenic (ALA) did not decreased C2C12 proliferation. Moreover, they found that when C2C12 were treated with EPA or DHA at the low concentrations (10 μM), the proliferation-inhibiting was not detected in the short term but could be easily observed after 3 days. These results suggested the inhibitory effects of EPA and DHA at high concentrations were not due to their cytotoxic effects.

Luo et al. [21] reported that very high concentrations of EPA (200–400 μM) inhibited C2C12 myotubes formation but induced myoblasts transdifferentiation to adipocytes. Hsueh et al. [30] reported that 50 μM EPA and DHA reduced C2C12 mytubes formation and downregulated genes associated with myogenesis (MRF4, MyoD, MyoG and Pax7) but upregulated genes associated with adipogenesis (aP2, c/EBPa/b, PPARg, CPT1b and FAT). Their results implied that EPA and DHA might reduce myogenesis and increase adipogenesis in myotube formation. Similarly, we found that EPA or DHA suppressed the mRNA expressions of MHC, myogenin and skeletal α-actin at low doses (6.25, 12.5 and 25 μM). The inhibitory effect was stronger in cells treated with 50 or 100 μM EPA or DHA. The protein levels of MHC and myogenin were also significantly reduced by 50 μM EPA or DHA treatments. These results suggested that EPA and DHA might exert an inhibitory effect on C2C12 differentiation at low concentrations. Nuclear receptor peroxisome proliferator-activated receptor gamma (PPARG) and peroxisome proliferator-activated receptor delta (PPARD) are critical positive regulators of adipogenesis [31,32]. PPARG and PPARD are both necessary and sufficient for adipocyte differentiation [33,34]. In addition, aP2, C/EBPα and Adipoq were also the essential genes related to adipogenesis [35,36,37]. In our study, EPA and DHA inhibited C2C12 differentiation within 48 h; however, the mRNA expressions of PPARG, PPARD, aP2 and Adipoq showed no significant change at this time, and C/EBPα showed decreased expression (Figure S2). We also performed oil red O lipid staining assays, and there were no detectable oil red O positive cells in the EPA or DHA treatments (Figure S2). These indicated that the impaired differentiation occurred in the early stage of the EPA or DHA treatment, which might be not accompanied by a transdifferentiation process.

In this study, we treated C2C12 myoblasts with 50 μM EPA or DHA for 48 h in proliferation condition, then investigated the gene expression changes by using RNA-sequencing. Overall, there were more DEGs in the EPA treatment group compared with DHA treatment. Function analysis indicated that the enriched GO terms of the downregulated DEGs in EPA or DHA treatment were associated with the skeletal muscle development. Interestingly, 50 μM EPA or DHA significantly reduced myogenin, IGF2 and some muscle-specific contractile protein genes during proliferation. Myogenin is crucial for regulating the muscle fibers maturation [2]. The muscle-specific contractile protein genes, such as Tnnt3, Mylpf, Mb and Myh3, are essential for the formation of the terminally differentiated myotubes [25]. The insulin-like growth factors (IGF-1 and IGF-2) play major roles in the regulation of skeletal muscle development, and both are locally expressed in muscle cells. Recent studies have demonstrated that IGF-2 upregulates its own gene expression during myogenesis and this auto-regulatory loop is critical for muscle differentiation [38,39]: The knockdown of IGF-2 inhibited myoblast differentiation [40]. In addition, IGF2 is expressed at higher levels in the fetus than postnatal stages and is believed to play an important role in very early fetal development and organogenesis [41]. Thus, these key genes suppressed by EPA and DHA might result in the inhibition of myoblasts differentiation.

ERK1/2 and Akt kinases play important roles in cell growth and differentiation. The inhibitory effect of DHA and EPA on lymphocyte proliferation was associated with a reduction in the IL-2-induced phosphorylation of ERK and Akt [20]. Denys et al. [42] found that EPA and DHA inhibited PMA-induced ERK1/2 phosphorylation in Jurkat T cells. The inhibition of ERK1/2 and Akt phosphorylation suppresses the proliferation and differentiation of myoblasts [43,44,45]. In the present study, both EPA and DHA inhibited ERK1/2 and Akt phosphorylation at 1 h after treatment. However, EPA and DHA had no effect on the phosphorylation of ERK1/2 at 48 h and 72 h after treatment (Figure S3), suggesting the depression of ERK1/2 phosphorylation at 1 h might not be the direct cause of the inhibition of C2C12 growth at 48−72 h. IGFs activate multiple intracellular signal transduction cascades, including the PI3K/Akt/mTOR and the MAPK/ERK cascade [46]. Through binding to the IGFs receptors, IGF-2 can stimulate ERK1/2 phosphorylation [47]. In cultured myoblast, growth factor deprivation induces the production of IGF-2, which in turn activates the IGF-1 receptor and a major downstream PI3K/AKT pathway to initiate the differentiation program [48]. Thus, in the current study, the reduced phosphorylation of Akt and ERK1/2 in C2C12 myoblast might be associated with the expression of IGF-2 suppressed by EPA and DHA.

## 5. Conclusions

In summary, our findings indicate that EPA and DHA have inhibitory effects on C2C12 myoblasts proliferation and differentiation and the phosphorylation of ERK1/2 and Akt. RNA sequencing revealed EPA and DHA downregulated muscle-related genes, including myogenin, IGF-2 and some muscle-specific contractile protein genes, which might result in the inhibitory effect of these n-3 PUFAs on myoblast myogenesis.

## Figures and Tables

**Table 1:** Primer sequences used for real-time PCR.

Gene	Forward (5′-3′)	Reverse (5′-3′)
*MHC*	CGCCCACCTGGAGCGGATGA	CTTGCGGTCCTCCTCGGTCTGGT
*Myogenin*	CGGTGGAGGATATGTCTGTTG	GGTGTTAGCCTTATGTGAATGG
*Skeletal α-actin*	CAGAGCAAGCGAGGTATCC	GTCCCCAGAATCCAACACG
*Myh3*	ATGAGCGGCGTGTTAAGGA	ATTGACTTGCGATTCTGCGATA
*Mylpf*	TTTCCATCTGGAGCTACTGC	ATAATGCCATCCCTGTTCTG
*Myoglobin*	CTCCTAAGTCCCAGTCCATTT	CACTCCCTCTAAGCAACCCT
*Tnnt3*	GCCCTCATTGACAGCCACTT	CTCCTCCGCCAATCTGTTCT
*IGF2*	CGCTTCAGTTTGTCTGTTCG	AGGTAGACACGTCCCTCTCG
*Pparg*	ATGGAGCCTAACTTTGAGTT	CAGCAGGTTGTCTTGGATG
*Ppard*	TTCAGAGGACCAGCCACAG	GGAGACAGCAAGAACAGGAG
*C/EBPα*	CGTCTAAGATGAGGGAGTCAGG	CAGATGGAGGAGCACAGAG
*aP2*	CGACAGGAAGGTGAAGAGCA	CGACAGGAAGGTGAAGAGCA
*Adipoq*	ATCATTATGACGGCAGCAC	CAGATGGAGGAGCACAGAG
*β-actin*	CAGCCTTCCTTCTTGGGTAT	TGGCATAGAGGTCTTTACGG

## Supplementary Materials

The following are available online at http://www.mdpi.com/2073-4425/10/1/64/s1, Figure S1: The cluster analysis and the KEGG analysis of the DEGs, Figure S2: The effects of EPA and DHA on the transdifferentiation of myoblasts to adipocytes, Figure S3: Effect of EPA and DHA on the phosphorylation levels of ERK1/2 in C2C12 myoblasts at 48 and 72 h after treatment. Table S1: The DEGs in C2C12 myoblasts treated with DHA or EPA treatment.
