# Supplementary material for: EPA and DHA Inhibit Myogenesis and Downregulate the Expression of Muscle-related Genes in C2C12 Myoblasts
Source: Genes (Basel). 2019 Jan 18;10(1):64. doi: 10.3390/genes10010064 (PMC6356802; doi:10.3390/genes10010064)
Supplement: Supplementary file 1 [file genes-10-00064-s001.zip › genes-429534-supplementary/Figure S1-3.docx]

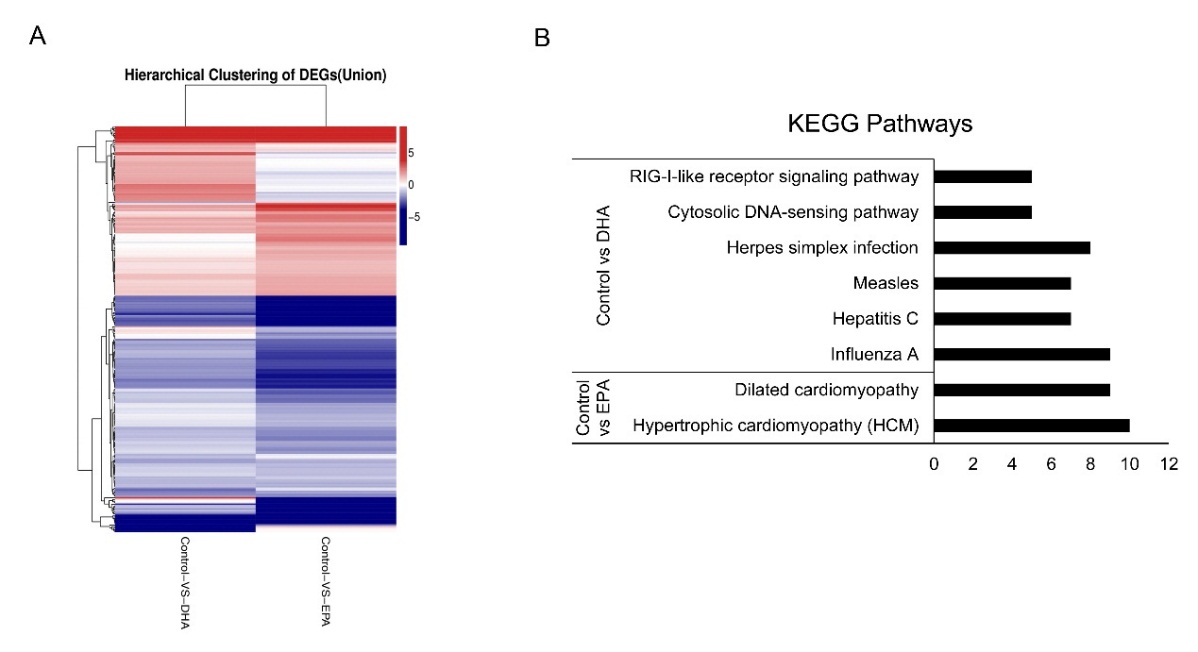


**Figure S1**. The cluster analysis and the KEGG pathways analysis of the DEGs. (A) The Union heat map of the DEGs. Gradient color barcode at the right top indicates log2 (FC) value. Each row represents a DEG and each column represents a condition pairwise. Red and blue represent an increase and decrease in expression, respectively. DEGs with similar fold-change values are clustered both at row and column level. (B) The enriched KEGG pathways of the DEGs in EPA treatment and DHA treatment. X axis represents number of DEGs. Y axis represents pathway.


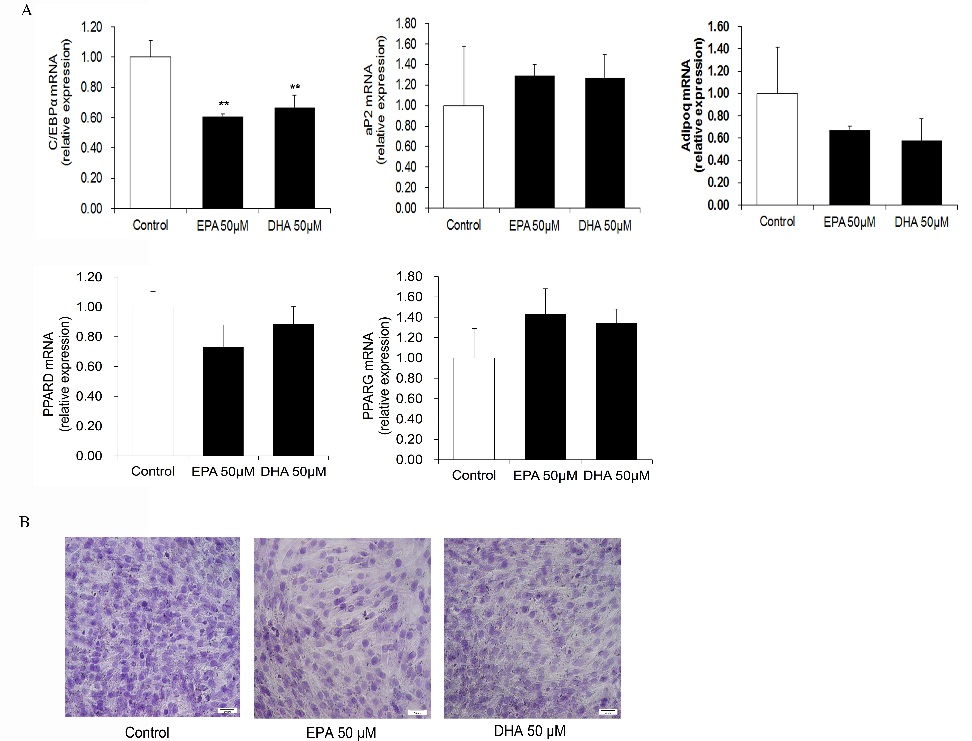


**Figure S2**. The effects of EPA and DHA on the transdifferentiation of myoblasts to adipocytes. (A) qRT-PCR was performed to examine the mRNA expression levels of aP2, C/EBPα, Adipoq, PPARG and PPARD in C2C12 cells treated without (control) or with EPA or DHA (50 μM) under differentiation conditions for 48 h. The data represent the mean ±S.D. from three independent experiments performed in duplicate. (B) Analysis of transdifferentiation by oil red O staining of C2C12 myoblasts. The C2C12 cells were treated with EPA or DHA without (control) or with EPA or DHA (50 μM) for 48 h before oil red O staining. Original magnifications 200 ×.


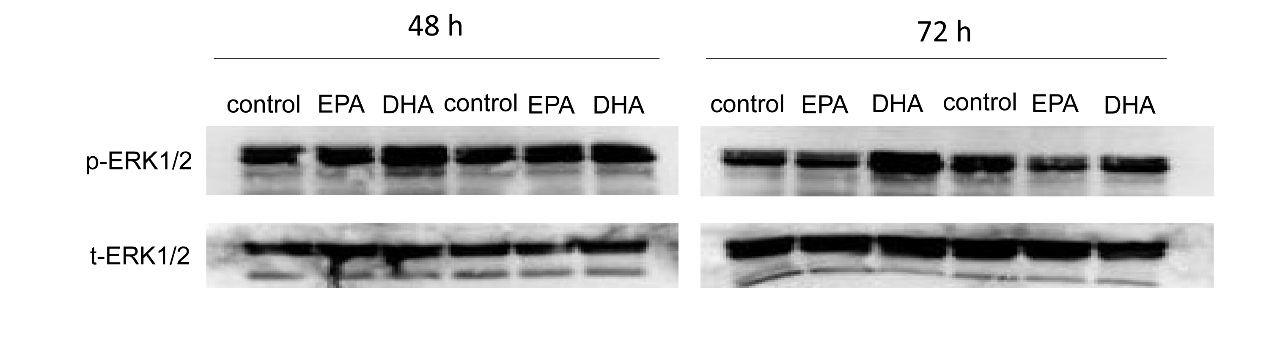


**Figure S3**. Effect of EPA and DHA on the phosphorylation levels of ERK1/2 in C2C12 myoblasts at 48 and 72 h after treatment.
